# Contextual influences on the development of obesity in children: A case study of UK South Asian communities^☆^

**DOI:** 10.1016/j.ypmed.2012.01.018

**Published:** 2012-03

**Authors:** Miranda Pallan, Jayne Parry, Peymane Adab

**Affiliations:** School of Health and Population Sciences, University of Birmingham, Edgbaston, Birmingham, B15 2TT, UK

**Keywords:** UK, South Asian, Child, Obesity, Food intake, Physical activity, Context

## Abstract

**Objective:**

An advocated approach to childhood obesity prevention research is the use of local community knowledge to inform intervention development. This paper demonstrates the value of accessing such local knowledge, and discusses how this information fits with existing conceptual models of childhood obesity.

**Methods:**

A series of 9 focus groups were run in 2007 with 68 local community stakeholders (including parents, school staff, community leaders and health and local government representatives) from 8 South Asian communities in Birmingham, UK to explore perceptions of factors contributing to the development of childhood obesity.

**Results:**

Perceptions of causal influences were grouped into several contexts, from the individual to the macro-level, that influence diet and physical activity. Specific cultural contextual data emerged that may explain decisions around physical activity and food intake of children within these communities. Assumptions made about South Asian communities were frequently contested.

**Conclusions:**

In order to truly understand the contextual influences on childhood obesity in target communities, it is necessary to access knowledge from local community members. Existing conceptual models of childhood obesity do not bring the role of cultural factors to the fore, but this context needs to be explicitly considered in the development of childhood obesity interventions.

## Highlights

► We examined contextual influences on childhood obesity in South Asian communities. ► We held focus groups with stakeholders from UK South Asian communities. ► Knowledge of context is critical for childhood obesity intervention development. ► Cultural influences on childhood obesity need to be understood in detail.

## Introduction

Childhood obesity is a global issue with an estimated 1 in 10 school-aged children being obese (Lobstein et al., 2004) but as yet, solutions to this problem are elusive. Childhood obesity prevention studies have at best, shown marginal short-term changes to weight status or behavioural outcomes (Bautista-Castano et al., 2004; Brown and Summerbell, 2009; Flodmark et al., 2006; Hardeman et al., 2000; Summerbell et al., 2005). A Cochrane review in 2005 called for a focus on intervention development, and the use of information from local community members to inform intervention design. This is coherent with the increasing focus on the development of complex health interventions, so that one can begin to understand how the various components and their interrelationships influence the target communities (Campbell et al., 2000; Craig et al., 2008).

This advocated approach to complex health interventions, including childhood obesity prevention programmes, necessitates a deep understanding of the determinants of the problem in the target communities. The importance of the relationship between context (e.g. socio-cultural structures and practices) and health, and in particular the relationship between context and individual health-related behaviours has been highlighted in recent years (Frohlich et al., 2001).

The work of Bronfenbrenner represents a major contribution to the theoretical understanding of the relationship between a child and the context within which they function. Bronfenbrenner proposed the Ecological Systems (ES) model, which depicts layers of contextual structures that influence a child, and in turn, these are influenced by the child's actions (Bronfenbrenner, 1977). These structures are termed the microsystems (the relationships between the child and their immediate environments, e.g. home, school), mesosystems (the interrelationships between these settings), exosystems (settings that have an indirect effect, e.g. neighbourhood), and macrosystems (cultural and societal values that are manifested in the micro-, meso- and exosystems). The ES model articulates the complexity and interactions of the contextual structures that a child is embedded in, and acknowledges the reciprocal nature of the relationships. The model is the basis for ecological health promotion models that attempt to move the focus away from individual behaviour change (McLeroy et al., 1988).

Bronfenbrenner's model has given rise to several conceptual models of childhood obesity. Davison and Birch's model depicts child weight status at the centre, surrounded by three concentric circles; child characteristics; parenting styles and family characteristics; and community, demographic and societal characteristics (Davison and Birch, 2001). A further example is the ‘Causal Web’ model for the development of obesity, proposed by the International Obesity Taskforce (IOTF), which schematically represents contextual influences on individual lifestyle ‘choices’ (Kumanyika et al., 2002). This model encompasses national and international factors (media and advertising, urbanisation etc.), akin to Bronfenbrenner's macrosystems, but does not acknowledge the reciprocity of relationships.

In this study, we report the findings from focus groups run with members of UK South Asian communities. South Asians are a particular target group for obesity prevention, as they have higher body fat than other ethnic groups, and are more vulnerable to the health consequences of obesity (Bhopal et al., 1999; Whincup et al., 2002; WHO expert consultation, 2004). The aim of the focus groups was to access key contextual data to inform the development of an obesity prevention programme targeting South Asian children. As part of the focus group process we explored participants' perceptions of causes of childhood obesity, and present this data here. We discuss the importance of accessing contextual information from communities targeted for intervention, and how the study findings fit with existing conceptual models of childhood obesity.

## Methods

The Birmingham healthy Eating and Active lifestyle for CHildren Study (BEACHeS) took place from 2006 to 2009 in a large multicultural UK city. The study used the theoretical, modelling and exploratory phases of the UK Medical Research Council framework for complex interventions (Campbell et al., 2000) to develop and pilot a childhood obesity prevention programme. Eight school communities with predominantly South Asian pupils (defined as Indian, Pakistani or Bangladeshi) participated in the study. All schools served materially disadvantaged populations. As part of the intervention development process focus groups with stakeholders were held, with the chief aim of generating and prioritising intervention ideas. Ethical approval was gained from the East Birmingham Local Research Ethics Committee.

### Participant recruitment

A stakeholder was defined as a local community member who had a connection to primary school-aged children. Stakeholder identity groups specified were; parents, teachers, school catering staff, other school support staff, healthcare professionals (e.g. school nurses), local authority representatives, prominent community members (e.g. school governors, religious leaders), leisure centre staff, and retail representatives.

Potential participants were purposively identified and recruited through participating schools. South Asian participants were actively sought as they were key informants (Mays and Pope, 1995). Participants received a letter, then a follow up telephone call. Parents with a first language other than English were approached through parent-link workers (school–family liaison staff). We aimed to recruit 6–8 participants per group.

### Focus groups

Focus groups were run as identity groups to enable discussion of shared experiences (Kitzinger, 1995). Two moderators (both British speaking females, one Iranian and one mixed British–Asian) ran all focus group sessions together. Participants attended two sessions. Participants completed a consent form and a questionnaire asking for demographic information. All groups were conducted in English, except for one Punjabi speaking group of parents, in which a parent-link worker interpreted. All sessions were audio-recorded.

The objectives of the first session were to explore perceptions of obesity and its causes in childhood, and generate ideas of ways to prevent childhood obesity within the local communities. The objective of session 2 was to prioritise obesity prevention ideas for inclusion in an intervention programme. First, participants' intervention ideas were recapped and intervention initiatives that had been evaluated in previous research were presented to participants in a handout. This information was derived from eight systematic reviews (Bautista-Castano et al., 2004; Doak et al., 2006; Flodmark et al., 2006; Hardeman et al., 2000; NHS Centre for Reviews and Dissemination, 2002; Sharma, 2006; Stice et al., 2006; Summerbell et al., 2005), encompassing 70 studies. The participants were asked to consider their own intervention ideas and those presented, and prioritise potential elements of an intervention programme in three stages. Focus group schedules are shown in Table 1. Sessions lasted1–2 h. Audio-recordings were transcribed verbatim.

### Analysis

In order to explore perceptions of the causes of childhood obesity, we undertook a thematic analysis. Data were initially coded into emergent themes using NVivo7 computer package. An iterative inductive process was undertaken to identify relationships between themes and distill broad theoretical concepts (Spencer et al., 2003). All transcripts were reviewed by the two moderators. Thematic coding was undertaken by one moderator, and emergent themes and relationships between them were reviewed by the second moderator.

## Results

We convened 9 focus groups over 5 months in 2007. There was unavoidable heterogeneity within some groups, including one where a school governor was among a parent group. However, the flow of discussion was comparable to other parent sessions. In total there were 68 participants. The majority were female (60, 88%). Of 55 participants disclosing ethnicity, 30 (55%) were from the three South Asian groups. (Table 2).

### Emergent themes on perceived causes of childhood obesity

The two overarching themes of influences on the development of childhood obesity to emerge are unhealthy food intake and lack of physical activity. These themes are consistent across a range of contexts which can be grouped into six areas; child, family, culture, school, local environment and macro-environment, although there is much fluidity between these. In terms of the wider environmental influences, most groups discussed the local environmental context and professional participants explicitly articulated the wider societal view, particularly the influence of food marketing. Parents also implicitly alluded to societal influences through their stories. For example, reference was made to media influences, the shift to sedentary lifestyles and the local abundance of fast food/takeaway shops. More proximal factors identified related to child and parental behaviours. For example, participants cited work commitments limiting parental time for food preparation and family activities, and unsafe local environments prompting parents to limit children's physical activity.

Whilst much data is widely applicable, some specific cultural contextual factors serve to explain particular health behaviours in South Asian communities. For example, extended families often live in one dwelling with hierarchical structures that give the grandmother control within the family and influence over the diets of the children. Additionally, older family members are often first generation immigrants who may have come from an environment where food is less plentiful, and so view food differently to others. These factors provide an explanation as to why ‘fat’ children are viewed as healthy, and why food is lavished on children as a sign of affection.

Another example comes from Islamic communities, which have a strong religious identity. Faith leaders have a central role in the community and a significant amount of time is spent at the mosque (place of worship). Children from age 5 are required to attend mosque daily after school, which has implications for food and physical activity behaviours; time to engage in after-school physical activities, time for evening meal preparation and consumption, and time for travel between school, home and mosque is limited. This leads to consumption of energy dense snacks and use of cars instead of walking. These examples illustrate the importance of understanding the cultural context. Unhealthy food and physical activity behaviours become a rational course of action when viewed within these contexts.

Several cultural stereotypes and assumptions made around South Asian communities were contested, for example, the perception that South Asians always cook with ghee (clarified butter) was contested by a South Asian community leader who believed that healthier oils are increasingly used to prepare traditional meals. The widely perceived view of disadvantaged communities having poor access to healthy foods was contested by some participants who believed that there was local availability of inexpensive fruit and vegetables. A further example is the challenging of the perception that South Asian children lack interest in sports. These examples emphasise the danger of relying on assumptions, and the importance of actively seeking a detailed understanding of the communities of interest.

**Fig. 1:**
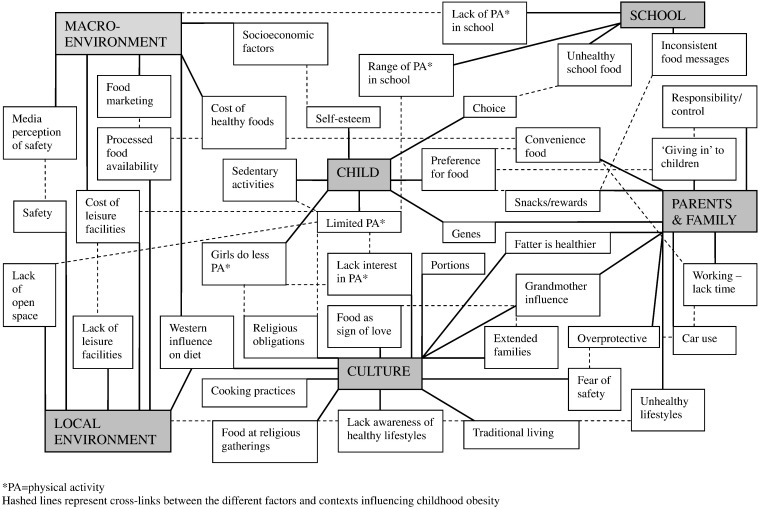
Schematic diagram of UK South Asian community stakeholders' perceived causes of childhood obesity (Birmingham, 2007). * PA =  physical activity. Hashed lines represent cross-links between the different factors and contexts influencing childhood obesity.

The themes emerging within the different contextual levels are presented in Table 3 with illustrating quotes. Crucially, the interrelationships between the different factors are numerous, multidirectional, and operate across the different contextual levels. Thus from the data we have built up a complex network of contextual factors contributing to the development of childhood obesity in UK South Asian communities (Fig. 1).

## Discussion

Overall, participants identified a broad range of contributors to childhood obesity, across multiple contextual levels. There was much focus on the role of parents and family, and many external influences on parents were identified.

The South Asian cultural context featured throughout all discussions. In addition to the influence of South Asian family structures, there was focus on traditional cooking practices, social and religious practices, and cultural and religious influences on physical activities. There was also a perception of a lack of awareness of healthy lifestyles in these communities. Acculturation was touched on by some participants, in terms of the changing diets within South Asian communities.

The findings of this study resonate with the perceptions of contributors to childhood obesity in various communities internationally. Themes such as child preference, sedentary activities, parental role models, constrained parental time, unhealthy school food, access to leisure facilities, fast food availability, food marketing and safety have been identified by communities across the globe (Hardus et al., 2003; Hesketh et al., 2005; Monge-Rojas et al., 2009; O'Dea, 2003; Power et al., 2010; Sonneville et al., 2009; Styles et al., 2007; Wilkenfield et al., 2007). One may conclude then that very different communities have similar causal influences on the development of childhood obesity. However, closer examination of the data reveals differences that are essential to understand when planning childhood obesity prevention. It is only by examining the particular community context that we can begin to understand why individuals take decisions to behave in a certain way.

A characteristic of South Asian communities is the central role of religious practices. Whilst this is not unique, understanding the precise nature of these is a prerequisite for successful intervention. To take a simple example, the provision of more after school clubs is unlikely to influence physical activity levels in a community where the majority of children attend mosque every day after school. The contestation of cultural stereotypes that emerged in this study further highlights the necessity of gaining a true understanding of the cultural context of communities targeted for intervention. Other studies have also drawn attention to cultural influences (Blixen et al., 2006; Monge-Rojas et al., 2009; Styles et al., 2007). In one focus group study of English and Spanish-speaking parents in the USA, the latter, but not the former group voiced that thinness was traditionally viewed as unhealthy (Sonneville et al., 2009). This understanding of the differing cultural contexts is crucial to successful childhood obesity intervention. Without this knowledge, we may miss the real opportunities for intervention.

Let us now consider how the study findings fit with the conceptual models of childhood obesity development. Participants articulated the complex and interlinking influences on childhood obesity. Whilst the greatest focus was on children and their families, the wider societal influences were discussed at local, national and international levels. Participants showed a sophisticated understanding of the reciprocity of influences across different contextual levels, for example, the relationship between parental safety fears and the media portrayal of unsafe local environments. The stakeholders' perceptions of childhood obesity causes therefore largely concur with existing conceptual models (Davison and Birch, 2001; Kumanyika et al., 2002). However, a central finding is the importance of the cultural context. Existing theoretical models do not explicitly consider this (Davison and Birch, 2001; Kumanyika et al., 2002), which is a potential weak point in application of such frameworks to analyse target communities. Hughes and DuMont argued for the use of focus groups to unlock the cultural knowledge of communities and facilitate development of conceptual frameworks (Hughes and DuMont, 1993). They emphasised that to impose a conceptual framework on a community risks omission of constructs that are central to their experiences. With this and the study findings in mind, we would advocate that the cultural context is made explicit in theoretical models of childhood obesity development. This would ensure that crucial information is not overlooked.

There were several limitations in this study. Focus groups often had a small number of participants and many did not attend both sessions, which may have limited discussion. However, a variety of stakeholders were recruited so a broad range of views were accessed. Few men participated, so the views expressed are largely from a female perspective. It is possible that different themes would have emerged had there been more male participants. This is a potential area for further exploration. This study explored South Asian community perceptions, and so we would not expect to generalise the findings to other communities. Nevertheless many emerging themes were similar to those found in other communities. Furthermore, the importance of the cultural context in the development of childhood obesity could be applied to any community. The problem with understanding the cultural context is that it may vary between neighbourhoods, religious groupings, or even families within the same community. Therefore, whilst some findings could be applied to all South Asians, some will only be relevant to specific groups.

In conclusion, the use of focus groups to access information from a range of community stakeholders has enabled us to construct a complex picture of the contextual influences acting on children. We have highlighted the importance of understanding cultural contextual influences on the development of childhood obesity, and the dangers of inaccurate assumptions. We suggest that cultural influences need to be explicitly articulated in conceptual models of childhood obesity development, as this will guide researchers to seek to understand this aspect of context when developing childhood obesity interventions.

## Conflict of interest statement

The authors have no competing interests to declare.

## Figures and Tables

**Table 1:** Schedules for first and second focus group sessions with UK South Asian community stakeholders (Birmingham, 2007).

Session 1: objectives	Session 2: objectives
• To explore understanding and perspectives on obesity/childhood obesity	• To present list of interventions from first focus group
• To explore ideas on childhood obesity causes (in the local setting)	• To present additional interventions from the literature
• To gather ideas on interventions to prevent childhood obesity	• To prioritise potential interventions

Session 1: process	Session 2: process

*1. Childhood overweight and obesity: is it a problem? Explore overweight and obesity, and related problems/advantages. Explore specifically perceptions of overweight/obesity in children.*	*1. Recap on interventions from previous discussion. Hand out a summary of interventions discussed at first session.*
▪ Exercise: “write on the slips of paper what word or short phrase comes to mind when you hear the words ‘overweight’ and ‘obese’”	▪ Does the group feel the summary is representative of discussions
▪ Share ideas on flip chart and explore	▪ Ask if anyone has further thoughts
*2. Causes of childhood obesity. Guide participants to think about the causes in the context of their local environments.*	*2. Presentation of additional interventions from research. Hand out list of intervention components from the literature. Put up board with participants' ideas and intervention components from the literature.*
▪ Explore why people think children become overweight and obese (probe on reasons behind poor diet and lack of physical activity)	▪ Ask what participants think of the intervention components identified from the literature (how these relate to their ideas)
▪ Why is childhood obesity increasing	*3. Prioritisation exercises. The group has to prioritise all ideas presented by moving through a series of stages, until they end up with a list of no more than 8 ideas to include in an intervention package.*
▪ Own experiences of influences on children's diets and physical activity	Stage 1
*3. Preventing childhood obesity. Get participants thinking about measures to encourage children to maintain healthy weights, especially in a local context.*	▪ Ask participants to discuss which of the interventions they think it are *most important* (*effective*) to include in an intervention package to encourage children to maintain a healthy weight
▪ Ask participants to think about what measures could help in encouraging children to maintain healthy weight	▪ Move the ideas from the original board to the ‘most important’ board, as directed by the group
▪ Hand out slips which can be used to note ideas	Stage 2
▪ Discussion, share ideas and note these on flipchart. Explore:	▪ Ask participants to now consider the interventions they think are the *most practical and most straightforward to deliver*
○ Similar concepts/measures	▪ Move interventions from the ‘most important’ board to the ‘practical’ board, as directed by the group
○ Barriers/enablers to implementing locally	Stage 3
○ Own experiences of measures that help change behaviour	▪ Ask participants to discuss and agree on up to *8 intervention components* to include in a package of activities
○ How participants might be involved in implementing	▪ Move interventions to the final board, directed by the group
	▪ Get group to reflect on the interventions that they have finally included and those they have left out

**Table 2:** UK South Asian community stakeholder focus group identities and participant characteristics (Birmingham, 2007).

Identity group	Participants at 1st session^a^ (female:male)	Participants at 2nd session^a^ (female:male)	Ethnicity^b^	Religious affiliation^b^
Parents (English speaking)	2:1	2:1 (one participant was a School Governor)	1Bangladeshi	3 Muslim
1 Pakistani	1 Christian
1 Yemeni	
1 British	
Parents (Punjabi speaking)	10:0	10:0	10 Pakistani	10 Muslim
Mothers of Pakistani origin (English speaking)	6:0	6:0	6 Pakistani	6 Muslim
Teachers	3:1	4:1	1 Indian	1 Sikh
3 White British	1Christian
1 White Canadian	1Bahai
1 Black Caribbean	1 No affiliation
Parents, school catering and school support staff	4:2	9:0	5 Indian	4 Sikh
2 Pakistani	3 Muslim
1 White British	1 Christian
1 White Irish	1 No affiliation
Catering and school support staff	7:1	7:1	2 Pakistani	2 Muslim
2 White British	2 Christian
	1 No affiliation
Community representatives including school governors	4:0	4:0	2 Indian	2 Sikh
2 White British	1 Christian
	1 No affiliation
Health representatives	8:0	6:0	6 White British	
1 Black Caribbean	
Local authority, retail and leisure representatives	1:3	1:3	5 White British	2 Christian
3 No affiliation

a Some participants attended only first or second sessions.

b Not all participants disclosed ethnicity or religious affiliation.

**Table 3:** Perceived influences on the development of childhood obesity: quotes from UK South Asian community stakeholder focus groups illustrating the emergent themes (Birmingham, 2007).

Contextual level	Influences on food intake	Influences on physical activity	Other influences
Child	Child preferences for unhealthy foods/snacks and expectation of choice: *“It's difficult to tell the children that, you know, vegetables are good for them, it's very difficult, because they don't know what's good for them, that's the problem, and they'll go for stuff which they'll like the taste of”* (mother) Low self-esteem: *“if they are having psychological difficulties like bullying or any other thing, they may be depressed and then they seek food and as a comfort”* (community leader)	Increased sedentary activities: *“But with kids, because their lifestyle is particularly more sedentary than it ever used to be in terms of sitting on computers and playing PSP's [games consoles] and all the other things where they just sit all day, then it's, that's the norm for them whereas the norm was as you were saying, going out to parks and running round and riding their bikes”* (school nurse)	
Family	‘Giving in’ to children's demands: *“it's a lot easier to let your child eat what it wants, it's hard work isn't it to say, no you are actually not going to have that today, you are going to sit down and have…”* (school nurse) Provision of unhealthy processed foods/snacks to children: *“parents meeting them at the gates with chocolate bars and candies that they eat walking along to home”* (teacher) Lack of time for meal preparation: *“It's more about the time though isn't it, with a lot of parents, they don't have the time to prepare and they don't seem to want to have the time to sit there and prepare the food, especially when you've been at work all day”* (retail representative) Lack of time for meal preparation contested: *“if we're talking about this area, no, because it's an extremely large Asian, we're talking about a South Asian population and traditionally the female would be in the house.”* (leisure centre manager) Parental food behaviours: *“So if we eat healthy then we'll feed our kids the same food, and if we eat junk food our kids will be eating the same food.”* (mother)	Safety concerns: *“I think security is a big thing. We don't let our kids out and play. I have to organise sports for them to actually play in a safe environment, which is a lot harder than like when I was a kid, you just, oh you just go out and play”* (mother) Parental time pressures: *“Bangladeshi people who work so they hardly find time to take children for a leisure activity”* (father) Car use: *“the traffic, and the roads that we have to cross, it's easier to put them in the car and go, it's quicker”* (mother) Inactive parental lifestyles: *“And also we have unhealthy home lifestyle in a lot of Pakistani, Bangladeshi, I don't know, maybe they smoke at home, and each of their parents work, they could not take the children out for vacation, and all the day the children are sitting in the home and watching TV.”* (father)	Biological influences: *“In some families, unfortunately, you know, the parents are big so the children are genetically big”* (mother) Role of genetics contested: *“They say this is a fallacy… because after the war there was nobody fat”* (catering manager) Parental responsibility: *“they [parents] don't take responsibility for the fact that their child [is overweight]”* (school nurse)
Culture	Traditional cooking practices: *“they're used to their traditional meal, and they're used to their way of cooking it and using the things that are, you know, like the fats, the ghee and butter”* (mother) Traditional cooking practices contested: *“people have started using olive oils now, rather than just pure butter and ghee, you know, clarified butter, so there is a shift from how it used to be”* (community leader) Family attendance at the mosque: *“a lot of the South Asian children in this area come home from school for half an hour and then they are at mosque until 7 or 8 o'clock at night, so there isn't time to sit down and eat a meal.”* (school nurse) Influence of extended families: *“the big families, you can't put them together, because everybody works, they go separate times to eat and things, but if you see like your own family, yeah, you can just put the food on the same I mean table so you can eat with your child and you can talk to him”* (mother) Central role of grandmothers: *“if my daughters have had one meal and then it was about 6 o'clock they ate I think, I went down to my mum's and when I came back at 8 o'clock they're saying they're hungry again, and so I'm saying ‘no you can't eat again because you ate at 6 so you should have something healthy, have a fruit now’ so my mother in law's like saying ‘no no no give them something to eat, they're hungry and you're shouting at them and you won't let them eat, when kids want food you give them food’”* (mother)	Contesting of perception that South Asian children lack interest in sports: *“[we need to] try and break this mould of the perception that children are lazy or the perception that a particular group of people don't like sport which very often is the case, with Asian culture, they think that they don't want to play football, they don't want to do a sport, well who says so, you know, and I mean that is just not true”* (school catering manager) Attitudes of South Asian communities regarding girls engaging in physical activity: *“they [South Asians] have this tendency, okay the boys can do anything, but the girls can't go swimming because they have to dress in front of boys”* (mother) Family attendance at the mosque: *“I think some, some communities they haven't got the time [to do physical activities], because they go to the mosque, they haven't got that time. As soon as they finish school, they have got activities [at the mosque] after school”* (school catering staff)	‘Thin’ viewed as unhealthy: *“and you get criticised as well if your children are thin. I mean, I get it all the time”* (mother)
School	Unhealthy school food and inconsistent food messages: *“That's something we still get resistance against when we require schools to run a whole school food policy, they still…the last thing that they want to get rid of is the sweet jar and the chocolate”* (local authority representative) Limited influence of schools: *“you can offer them [the children] this balance at school, but as soon as they go out, everything is cooked with you know, butter or fat.”* (teacher)	Lack of physical activity in the school day: *“I don't think they get enough exercise full stop in schools anyway, even in the curriculum… they're just paying lip service in my opinion, exercise and stuff, especially junior schools and infant schools”* (leisure centre manager) Emphasis on competitive sports: *“I think there is also an emphasis through the schools on traditional sports, which I don't think appeals if you are not a sporty person”* (community leader)	
Local and macro-environment	Access to convenience and fast foods: *“I see a lot of young kids, if a takeaway is near their house, they will walk round…”* (mother) Western influence on traditional diets: *“they are losing sometimes, some of the South Asians, some of the best things of their diet and getting things like Karachi fried chicken [a South Asian fast food outlet] and you know which is local to us, so they are taking the worst of what you would say, I don't know what you would call it, the British diet”* (community leader) Marketing of unhealthy food: *“my daughter, from a very early age, saw adverts on TV and when we used to go shopping she wants that, and she wants that, and I think it's as simple as that really, and it's taken in”* (local authority representative) Expense of healthy food: *“the healthier foods are dearer, you are paying a vast amount of money for good food, whereas junk food is much cheaper”* (catering staff) Expense of healthy food contested: *“Mum says people think about money as well, the fact that on takeaways you are spending a lot of money and like if you were to buy for that price the good foods, you know you get value for money, you feed more people.”* (Interpreter translating for Punjabi speaking mother)	Lack of accessible open space: *“I have certainly been shocked in the area that there is not a huge amount of green space where I work, but there are little bits of park and I have yet to see a young family in there. I have seen teenagers in there and I have seen men with horrible nasty looking dogs, but I haven't seen the young families”* (community leader) Cost of and lack of local leisure facilities: *“I don't think there is enough outreach work going on within the community, from the Council to the community in letting people know what's available. Again I don't think there is very much available in the area”* (mother) Physical activity as part of daily life: *“What it is, is that nowadays to stay a healthy weight, it requires a lot more effort than it did in the old days when we had to use the mangle and we had to walk every way, it's the effort that's changed”* (dietician) Media portrayal of an unsafe environment: *“the press has made out as if there's a weirdo on every corner, you know…and the tragedy is there are a few incidents, and then suddenly nowhere in the country is safe”* (leisure centre manager)	Socioeconomic issues taking precedence over healthy lifestyles: *“I suppose therefore if you are, I don't know poor or whatever, working class, or from… I don't know what the new terminology is, but …then are you going to be sitting there worrying about what your food intake is and whether you've got down to the gym that day, or are you going to be worried about whether you're going to pay your heating bill, whether your electric's about to switch itself off”* (retail representative)

## References

[bb0005] Bautista-Castano I., Doreste J., Serra-Majem L. (2004). Effectiveness of interventions in the prevention of childhood obesity. Eur. J. Epidemiol..

[bb0010] Bhopal R., Unwin N., White M. (1999). Heterogeneity of coronary heart disease risk factors in Indian, Pakistani, Bangladeshi, and European origin populations: cross sectional study. B.M.J..

[bb0015] Blixen C.E., Singh A., Thacker H. (2006). Values and beliefs about obesity and weight reduction among African American and Caucasian women. J. Transcult. Nurs..

[bb0020] Bronfenbrenner U. (1977). Toward an experimental ecology of human development. Am. Psychol..

[bb0025] Brown T., Summerbell C. (2009). Systematic review of school-based interventions that focus on changing dietary intake and physical activity levels to prevent childhood obesity: an update to the obesity guidance produced by the National Institute for Health and Clinical Excellence. Obes. Rev..

[bb0030] Campbell M., Fitzpatrick R., Haines A. (2000). Framework for design and evaluation of complex interventions to improve health. B.M.J..

[bb0035] Craig P., Dieppe P., Macintyre S. (2008). Developing and evaluating complex interventions: the new Medical Research Council guidance. B.M.J..

[bb0040] Davison K.K., Birch L.L. (2001). Childhood overweight: a contextual model and recommendations for future research. Obes. Rev..

[bb0045] Doak C.M., Visscher T.L., Renders C.M. (2006). The prevention of overweight and obesity in children and adolescents: a review of interventions and programmes. Obes. Rev..

[bb0050] Flodmark C.E., Marcus C., Britton M. (2006). Interventions to prevent obesity in children and adolescents: a systematic literature review. Int. J. Obes..

[bb0055] Frohlich K.L., Corin E., Potvin L. (2001). A theoretical proposal for the relationship between context and disease. Sociol. Health. Illn..

[bb0060] Hardeman W., Griffin S., Johnston M. (2000). Interventions to prevent weight gain: a systematic review of psychological models and behaviour change methods. Int. J. Obes..

[bb0065] Hardus P.M., van Vuuren C.L., Crawford D. (2003). Public perceptions of the causes and prevention of obesity among primary school children. Int. J. Obes..

[bb0070] Hesketh K., Waters E., Green J. (2005). Healthy eating, activity and obesity prevention: a qualitative study of parent and child perceptions in Australia. Health Promot. Int..

[bb0075] Hughes D., DuMont K. (1993). Using focus groups to facilitate culturally anchored research. Am. J. Community Psychol..

[bb0080] Kitzinger J. (1995). Qualitative research: introducing focus groups. B.M.J..

[bb0085] Kumanyika S., Jeffery R.W., Morabia A. (2002). Obesity prevention: the case for action. Int. J. Obes..

[bb0090] Lobstein T., Baur L., Uauy R. (2004). Obesity in children and young people: a crisis in public health. Obes. Rev..

[bb0095] Mays N., Pope C. (1995). Qualitative research: rigour and qualitative research. B.M.J..

[bb0100] McLeroy K.R., Bibeau D., Steckler A. (1988). An ecological perspective on health promotion programs. Health Educ. Behav..

[bb0105] Monge-Rojas R., Garita-Arce C., Sanchez-Lopez M. (2009). Barriers to and suggestions for a healthful, active lifestyle as perceived by rural and urban Costa Rican adolescents. J. Nutr. Educ. Behav..

[bb0110] NHS Centre for Reviews and Dissemination (2002). The Prevention and Treatment of Childhood Obesity.

[bb0115] O'Dea J. (2003). Why do kids eat healthful food? Perceived benefits of and barriers to healthful eating and physical activity among children and adolescents. J. Am. Diet. Assoc..

[bb0120] Power T.G., Bindler R.C., Goetz S. (2010). Obesity prevention in early adolescence: student, parent, and teacher views. J. Sch. Health.

[bb0125] Sharma M. (2006). School-based interventions for childhood and adolescent obesity. Obes. Rev..

[bb0130] Sonneville K.R., La Pelle N., Taveras E.M. (2009). Economic and other barriers to adopting recommendations to prevent childhood obesity: results of a focus group study with parents. B.M.C. Pediatr..

[bb0135] Spencer L., Ritchie J., O'Connor W., Ritchie J., Lewis J. (2003). Analysis: practices, principles and processes. Qualitative Research Practice: A Guide for Social Science Students and Researchers.

[bb0140] Stice E., Shaw H., Marti C.N. (2006). A meta-analytic review of obesity prevention programs for children and adolescents: the skinny on interventions that work. Psychol. Bull..

[bb0145] Styles J.L., Meier A., Sutherland L.A. (2007). Parents' and caregivers' concerns about obesity in young children. Fam. Community Health..

[bb0150] Summerbell C.D., Waters E., Edmunds L.D. (2005). Interventions for preventing obesity in children. Cochrane Database of Systematic Reviews: Reviews 2005 Issue 3.

[bb0155] Whincup P.H., Gilg J.A., Papacosta O. (2002). Early evidence of ethnic differences in cardiovascular risk: cross sectional comparison of British South Asian and white children. B.M.J..

[bb0160] WHO expert consultation (2004). Appropriate body-mass index for Asian populations and its implications for policy and intervention strategies. Lancet.

[bb0165] Wilkenfield R., Pagnini D., Booth M. (2007). The Weight of Opinion: Perceptions of School Teachers and Secondary Students on Child and Adolescent Overweight and Obesity.

